# QTL mapping and candidate gene analysis for yield and grain weight/size in Tartary buckwheat

**DOI:** 10.1186/s12870-022-04004-x

**Published:** 2023-01-26

**Authors:** Ruiyuan Li, Zhengfeng Chen, Ran Zheng, Qingfu Chen, Jiao Deng, Hongyou Li, Juan Huang, Chenggang Liang, Taoxiong Shi

**Affiliations:** 1 Key Laboratory of Information and Computing Science of Guizhou Province, Guizhou Normal University, Guiyang, 550001 Guizhou China; 2 Research Center of Buckwheat Industry Technology, Guizhou Normal University, Guiyang, 550001 Guizhou China

**Keywords:** Tartary buckwheat, Yield, Grain weight/size, QTL, SNP/InDel variation, Candidate gene

## Abstract

**Background:**

Grain weight/size influences not only grain yield (GY) but also nutritional and appearance quality and consumer preference in Tartary buckwheat. The identification of quantitative trait loci (QTLs)/genes for grain weight/size is an important objective of Tartary buckwheat genetic research and breeding programs.

**Results:**

Herein, we mapped the QTLs for GY, 1000-grain weight (TGW), grain length (GL), grain width (GW) and grain length–width ratio (L/W) in four environments using 221 recombinant inbred lines (XJ-RILs) derived from a cross of 'Xiaomiqiao × Jinqiaomai 2'. In total, 32 QTLs, including 7 for GY, 5 for TGW, 6 for GL, 11 for GW and 3 for L/W, were detected and distributed in 24 genomic regions. Two QTL clusters, *qClu-1-3* and *qClu-1-5*, located on chromosome Ft1, were revealed to harbour 7 stable major QTLs for GY (*qGY1.2*), TGW (*qTGW1.2*), GL (*qGL1.1* and *qGL1.4*), GW (*qGW1.7* and *qGW1.10*) and L/W (*qL/W1.2*) repeatedly detected in three and above environments. A total of 59 homologues of 27 known plant grain weight/size genes were found within the physical intervals of *qClu-1-3* and *qClu-1-5*. Six homologues, *FtBRI1*, *FtAGB1*, *FtTGW6*, *FtMADS1*, *FtMKK4* and *FtANT*, were identified with both non-synonymous SNP/InDel variations and significantly differential expression levels between the two parents, which may play important roles in Tatary buckwheat grain weight/size control and were chosen as core candidate genes for further investigation.

**Conclusions:**

Two stable major QTL clusters related to grain weight/size and six potential key candidate genes were identified by homology comparison, SNP/InDel variations and qRT‒qPCR analysis between the two parents. Our research provides valuable information for improving grain weight/size and yield in Tartary buckwheat breeding.

**Supplementary Information:**

The online version contains supplementary material available at 10.1186/s12870-022-04004-x.

## Background

Tartary buckwheat (*Fagopyrum tataricum* (L.) Gaertn.) is a widely cultivated pseudocereal in many mountain regions of Himalayan countries, China, Korea, Japan, Russia, the USA, Ukraine and Europe [1]. Tartary buckwheat has grains similar to true cereals in physical appearance and high starch content [2]; furthermore, its grains have high nutritional value and health care function because of high levels of crude protein [1], resistant starch [3], essential aminoacids and trace elements [4–6], rutin with multiple phenolic hydroxyl groups [7–9], dietary fiber and vitamins [2, 10], which have been identified as “Future Smart Foods” by the Food and Agriculture Organization (FAO) [10]. Tartary buckwheat grains are used to produce buckwheat rice, health tea, noodles, porridge, bread, pancakes, sprouts for salads and smoothies, and even drinks [11]. However, the grain yields of current Tartary buckwheat cultivars are low (1700 to 2500 kg∙hm^−2^) [12], and cannot meet the increasing market demand. Thus, the improvement in yield potential has been the major task in Tartary buckwheat breeding.

Grain yield (GY) is a complex and quantitatively inherited trait associated with multiple yield-related traits. Thousand grain weight (TGW) is one of the most important constituent factors of crop yield. Generally, TGW exhibited a significant positive contribution to Tartary buckwheat yield [13–16]. Grain size, including grain length (GL), grain width (GW) and length-to-width ratio (L/W), plays a key role in determining grain yield by affecting TGW. It has been reported that TGW is extremely significantly positively correlated with GL and GW and significantly negatively correlated with L/W in Tartary buckwheat [17]. Grain weight/size also influences grain protein and starch content [18, 19], appearance quality and consumer preference [20]. Therefore, genetic study of grain weight/size of Tartary buckwheat will help to breed high-yield and high-quality varieties and increase the commercial value of buckwheat rice.

Given the important influence of grain weight/size on yield and quality formation, studies of quantitative trait locus (QTL) mapping, gene cloning and functional verification for grain size/weight genes have gradually deepened in the modern plant Arabidopsis [21] and major cereal crops in the last decade [21–24]. However, genetic studies of buckwheat grain-related traits have just started along with the release of the draft genome [25] in recent years, which mainly focused on transcriptome analysis during Tartary buckwheat grain development to give insight into its transcriptional dynamics and find candidate genes that may be involved in grain development [26–30]. The grain weight/size of Tartary buckwheat has high additive effects and broad sense heritability [17], indicating the presence of potential stable QTLs. However, up to date, very few QTLs associated with grain-related traits have been identified in Tartary buckwheat. Three major QTLs or TGW were first detected on chromosomes Ft1 and Ft4 using an RILs population in our previous study [31]. Three candidate genes significantly correlated with TGW and GW were identified on chromosomes Ft1, Ft3 and Ft4 by genome-wide association studies (GWASs) [32]. A greater understanding of genomic information and QTLs underlying grain-related traits is essential for gene discovery and marker-assisted selection in improving Tartary buckwheat yield. In this study, QTL mapping for GY, TGW, GL, GW and L/W was performed in four environments using the availability of a high-density SNP linkage map developed from the RILs population of ‘Xiaomiqiao × Jinqiaomai 2’ (XJ-RILs population) [31], and candidate genes for stable major QTLs were predicted by combining homology comparison, sequence variations and qRT‒PCR analysis between the two parents. The QTLs and candidate genes identified in this study can facilitate future molecular breeding programs to improve grain weight/size and yield in Tartary buckwheat.

## Materials and methods

### Genetic materials

Two contrasting parents in terms of yield, grain weight and size were crossed to generate 221 recombinant inbred lines (XJ-RILs population) [31]. The maternal parent Xiaomiqiao is a Rice-Tartary type with small grains, thin and loose hull, long vegetative period and low yield, whereas the paternal parent Jinqiaomai 2 is a Tartary buckwheat type with large grains, thick and adherent hull, short vegetative period and high yield. Among the XJ-RILs, the grain hull of 79 lines were the ‘Rice’ type, and 142 lines were the ‘non-Rice’ type.

### Field experiment and trait evaluation

The XJ-RILs population along with the two parents were planted in four environments in Guizhou Province: Changshun (26°27' N, 106°39' E) in August 2017 and Baiyi (26°64' N, 106°63' E) in August 2018, 2019 and 2020. The average temperature was 21.65 °C/14.48 °C, 21.62 °C/14.66 °C, 24.98 °C/16.55 °C and 19.34 °C/13.48 °C, and the amount of precipitation was 532.9 mm, 309.3 mm, 294.4 mm and 468.6 mm during the growing period in 2017, 2018, 2019 and 2020, respectively (Additional file 1: Table S1). The experimental field was set up with a randomized block design with three replications. Each experimental plot consisted of three rows with 2.0 m in length and 0.33 m between rows, with approximately 100 grains per row. Field management was based on the local practices throughout the growth period.

After maturity, each plot was harvested and threshed separately by hand. GY was calculated based on the grain dry weight of each plot. Fully filled dry grains were used for determining TGW, GL, GW and L/W using a Wanshen SC-A seed detector (Hangzhou Wanshen Detection Technology Co., Ltd.). The mean values of each trait over three replications were used to analyse data for the individual environment. Broad-sense heritability was calculated following the equation described by Gu et al. [33].

### QTL analysis

An ultrahigh density genetic map for the XJ-RILs population was exhibited with 4,151 bin markers comprising 122,185 SNPs in our previous study [31]. QTL mapping and the estimation of QTL effects for the tested traits were performed by the composite interval mapping (CIM) model in Windows QTL Cartographer v 2.5 software (https://brcwebportal.cos.ncsu.edu/qtlcart/WQTLCart.htm) with default settings. To identify an accurate significance threshold for each trait, an empirical threshold was determined for CIM using 1000 permutations. The locus with an LOD value over the empirical threshold determined by 1000 permutations was considered a QTL, and the confidence interval was estimated using the 2 LOD-drop method. QTLs for the same trait detected from different environments with overlapping confidence intervals and the same donor for corresponding alleles were predicted to be the same QTL [33–35]. QTLs identified in multiple environments and explained more than 10% of the phenotypic variance were considered major QTLs. QTLs for different traits with overlapping confidence intervals were considered to be a QTL cluster.

### Candidate gene prediction

The physical intervals of major QTL clusters were aligned to the Tartary buckwheat reference genome [25] to identify the corresponding genes. To predict the candidate genes regulating Tartary buckwheat grain weight/size, a blastn search, with default parameters setting except the e-value < 10^–6^, was conducted within the intervals of major QTL clusters to find the homologues of known plant grain weight/size genes [21, 36]. The first reported gene of the blastn search was considered to be the homologue to the searched gene and its function was assigned.

### SNP/InDel variations and effect analysis of candidate genes

SNPs/InDels located in the genes within the physical intervals of the major QTL clusters were extracted from our previous study [31]. The effect of the extracted SNP/InDel (synonymous, stopgain, stoploss and splicingloss) was estimated by annovar (https://annovar.openbioinformatics.org/en/latest/) in the gene-based annotation model using the default setting and the gene annotation of Pinku1 (http://www.mbkbase.org/Pinku1/).

### Expression analysis of candidate genes by qRT‒PCR

qRT‒PCR analysis was performed to analyse the expression levels of seven homologues of known grain weight/size genes with SNP/InDel variations located in exon or splicing events. The reference cDNA sequences of these genes were obtained from the Tartary buckwheat genome sequence database. The qRT‒PCR primers were designed using Primer 5.0 software according to the reference cDNA sequences (Additional file 1: Table S2).

Grains after pollination (5, 10 and 15 days) were collected from Xiaomiqiao and Jinqiaomai 2. RNA isolation and cDNA preparation of candidate genes were performed as described in [31]. qRT‒PCR was conducted using SuperReal PreMix Plus (SYBR Green) (Tiangen, Beijing, China) on a CFX96TM Real-Time PCR Detection System (Bio-Rad, USA). Each sample was analysed in triplicate. The relative expression change of each gene was calculated using the 2^−∆∆Ct^ method.

## Results

### Phenotypic variations in yield and grain weight/size

The phenotypic values of GY, TGW, GL, GW and L/W determined in the paternal parent Jinqiaomai 2 of the mapping population were consistently extremely significantly higher than those in the maternal parent Xiaomiqiao, except for in 2017, where GW showed no significant difference between the two parents. In the XJ-RILs populatin, the values of the five tested traits of the ‘non-Rice’ type were significantly or extremely significantly higher than those of the ‘Rice’ type except GL, GW and L/W in 2020. The mean GYs of ‘Xiaomiqiao’ and ‘Rice’ type lines in 2020 were dramatically lower than those in other three environments, mainly due to the longer vegetative period of ‘Xiaomiqiao’ and a part of ‘Rice’ type lines but sustained low temperature (less than 10 ℃) at the initial maturity stages in 2020 (Additional file 1: Table S1). Relatively more variation was observed for the following three traits: GY, TGW and L/W (Table 1 and Additional file 2: Fig. S1). Wide range and continuous distributions were observed for all tested traits both in the ‘non-Rice’ type lines and the ‘Rice’ type lines (Additional file 2: Fig. S1), indicating that these traits were controlled by multiple loci. Transgressive segregation was observed for the five tested traits in all environments, except for GL and L/W in 2017, GL, GW and GY in 2020 (Table 1). TGW, GL and L/W showed a more-or-less bimodal distribution, suggesting the presence of potential major QTLs in the XJ-RIL population (Additional file 2: Fig. S1).

**Table 1:** Phenotypic distribution of five grain-related traits in the XJ-RILs population derived from the cross of ‘Xiaomiqiao × Jinqiaomai 2’ in four environments

Trait	Environment	Parents		XJ-RILs population								
				Rice type			Non-Rice type			Skewness	Kurtosis	CV%
		Xiaomiqiao	Jinqiaomai 2	Mean	Min	Max	Mean	Min	Max			
TGW (g)	2017	16.78 ± 0.82	20.08 ± 0.56**	17.16 ± 2.18	12.88	22.92	21.24 ± 1.73**	16.57	27.30	-0.46	-0.25	14.0
	2018	14.52 ± 1.41	20.65 ± 0.89**	14.90 ± 1.91	12.01	22.17	19.98 ± 1.79**	14.08	23.69	-0.32	-1.27	16.8
	2019	12.34 ± 0.26	19.40 ± 0.44**	13.99 ± 1.94	11.17	20.41	17.56 ± 1.51**	12.53	21.12	-0.25	-0.92	14.8
	2020	12.96 ± 0.35	20.71 ± 1.38**	15.99 ± 1.95	11.35	20.62	17.25 ± 1.93**	12.53	21.98	-0.03	-0.32	11.9
GL (mm)	2017	4.60 ± 0.14	5.37 ± 0.11**	4.39 ± 0.30	3.90	5.15	4.68 ± 0.37**	4.00	5.35	0.21	-1.15	8.1
	2018	4.46 ± 0.30	5.24 ± 0.31**	4.52 ± 0.38	4.01	5.57	4.98 ± 0.40**	4.11	5.64	-0.01	-1.21	9.3
	2019	3.81 ± 0.03	5.03 ± 0.53**	4.33 ± 0.36	3.77	5.42	4.71 ± 0.34**	3.85	5.27	-0.13	-0.90	8.5
	2020	4.27 ± 0.09	6.39 ± 0.19**	5.86 ± 0.44	5.00	6.55	5.88 ± 0.39	5.05	6.60	-0.02	-0.84	6.3
GW (mm)	2017	2.99 ± 0.07	2.94 ± 0.10	2.95 ± 0.11	2.60	3.30	3.06 ± 0.13**	2.70	3.43	0.32	0.40	4.6
	2018	2.89 ± 0.12	3.13 ± 0.16**	2.97 ± 0.12	2.75	3.35	3.17 ± 0.15**	2.80	3.54	0.37	-0.24	5.7
	2019	2.57 ± 0.03	2.87 ± 0.03**	2.84 ± 0.13	2.56	3.17	3.02 ± 0.15**	2.69	3.44	0.11	-0.21	5.4
	2020	2.87 ± 0.06	3.36 ± 0.06**	3.30 ± 0.20	2.92	3.70	3.37 ± 0.20	2.91	3.86	0.26	-0.70	5.6
L/W	2017	1.54 ± 0.06	1.83 ± 0.08**	1.49 ± 0.12	1.30	1.75	1.54 ± 0.16*	1.27	1.82	0.20	-1.34	10.0
	2018	1.55 ± 0.05	1.70 ± 0.07**	1.54 ± 0.14	1.33	1.79	1.59 ± 0.18*	1.27	1.86	-0.02	-1.53	10.8
	2019	1.50 ± 0.01	1.76 ± 0.02**	1.53 ± 0.14	1.34	1.79	1.58 ± 0.16*	1.29	1.83	-0.06	-1.58	10.1
	2020	1.50 ± 0.02	1.93 ± 0.07**	1.80 ± 0.18	1.46	2.23	1.80 ± 0.19	1.39	2.16	0.05	-1.23	10.2
GY (kg/ha)	2017	1032.4 ± 161.5	1346.0 ± 246.9*	1458.0 ± 380.7	561.2	2243.9	1843.7 ± 378.3**	851.2	2785.4	-0.04	-0.19	25.2
	2018	979.1 ± 281.1	1262.3 ± 197.1**	1240.3 ± 350.8	561.2	2141.9	1471.5 ± 366.9**	505.3	2502.1	0.06	0.19	27.0
	2019	1349.5 ± 403.3	1897.1 ± 378.6**	1651.9 ± 352.5	754.9	2437.8	1934.9 ± 322.8**	714.8	2549.6	-0.38	-0.31	21.1
	2020	31.4 ± 4.5	2506.2 ± 721.6**	734.9 ± 529.0	138.5	2318.5	1257.1 ± 676.3**	107.6	3656.9	0.62	-0.51	64.3

*TGW* 1000-grains weight, *GL* Grain length, *GW* Grain width, *L/W* Grain length-to-width ratio, *GY* Grain yield, *CV%* Coefficient of variation

^**^ and * indicate significant difference between the two parents, and between the 'Rice' type lines and the 'non-Rice' type lines at *p* < 0.01 and *p* < 0.05, respectively

ANOVA showed that genotype, year and genotype × year interaction effects were significant for all five tested traits at *P* < 0.001 (Additional file 1: Table S3). The broad sense heritability of TGW, GL, GW and L/W ranged from 79.5% to 84.7%, which was approximately two times higher than that of GY (37.8%) (Additional file 1: Table S3).

The Pearson’s correlation coefficients between the five tested traits are shown in Table 2. An extremely significant positive correlation was observed between GY and TGW in all environments. GY was significantly positively correlated with GL except in 2020 and significantly positively correlated with GW except in 2017. TGW was extremely significantly positively correlated with GW and GL in all environments, except for GL in 2020. Compared with GL, higher Pearson’s correlation coefficients were observed between TGW and GW except in 2019.

**Table 2:** Pearson’s correlation coefficients among traits in the XJ-RILs population derived from the cross of ‘Xiaomiqiao × Jinqiaomai 2’ in four environments

Traits	Environment	TGW	GL	GW	L/W
GY	2017	0.315^**^	0.192^**^	0.092	0.113
	2018	0.337^**^	0.265^**^	0.190^**^	0.124
	2019	0.395^**^	0.160^*^	0.344^**^	-0.035
	2020	0.445^**^	-0.002	0.169^*^	-0.087
TGW	2017		0.430^**^	0.447^**^	0.152^*^
	2018		0.587^**^	0.721^**^	0.138^*^
	2019		0.588^**^	0.539^**^	0.216^**^
	2020		0.024	0.397^**^	-0.205^**^
GL	2020			-0.145^*^	0.893^**^
	2018			-0.011	0.864^**^
	2019			0.040	0.833^**^
	2020			-0.515^**^	0.891^**^
GW	2017				-0.567^**^
	2018				-0.509^**^
	2019				-0.491^**^
	2020				-0.845^**^

*GY* Grain yield, *TGW* 1000-grains weight, *GL* Grain length, *GW* Grain width, *L/W* Grain length-to-width ratio

^**^ and * indicate significant correlation at *p* < 0.01 and *p* < 0.05, respectively

### QTL mapping for yield and grain weight/size in four environments

**Fig. 1:**
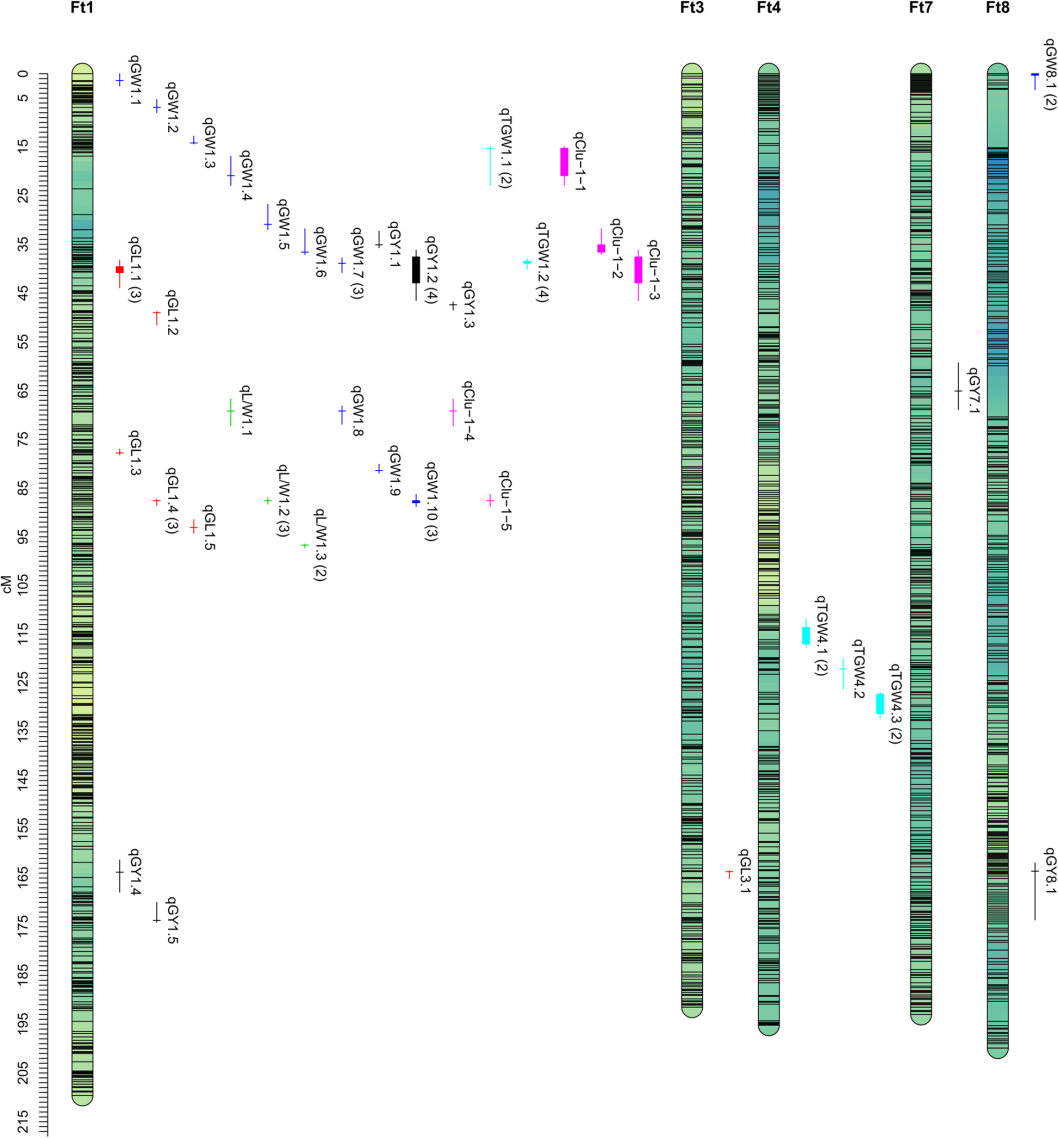
The distribution of the QTLs for grain yield (GY), 1000-grain weight (TGW), grain length (GL), grain width (GW), grain length–width ratio (L/W) and QTL clusters detected in the XJ-RILs population derived from the cross of ‘Xiaomiqiao × Jinqiaomai 2’ in four environments. The red, green, blue, black, cyan and magenta lines represent QTLs for GL, L/W, GW, GY, TGW and QTLs clusters, respectively. The horizon lines indicate the peak position, and the vertical lines indicate the confidence interval of QTLs. The interval of the vertical filled rectangle represents the minimal and maximal peak positions of QTLs repeatedly detected in different environments, and the number in brackets gives the number of environments in which the QTL detected

A total of 53 significant QTLs were identified for the five tested traits across four environments. The LOD values of all QTLs ranged from 3.30 to 43.59, explaining 3.41% to 58.79% of the phenotypic variance (*R*^2^) (Table 3). QTLs for the same trait detected in different environments were considered to be the same if the confidence intervals overlapped and the positive alleles were provided by the same parent. Finally, 32 QTLs for the five tested traits were obtained and distributed on chromosomes Ft1, Ft3, Ft4, Ft7 and Ft8 (Fig. 1). Globally, the largest number of QTLs (25) was detected on chromosome Ft1. Among the 32 QTLs, 12 QTLs repeatedly detected in two or more environments were regarded as multi-environmental QTLs, and the other 20 QTLs detected in only one environment were considered environment-specific QTLs (Table 3 and Fig. 1). Both parental lines contributed the favourable alleles depending on the QTLs (20 by ‘Jinqiaomai 2’ and 12 by ‘Xiaomiqiao’) (Table 3).

**Table 3:** QTLs detected for five grain-related traits in the XJ-RILs population derived from the cross of ‘Xiaomiqiao × Jinqiaomai 2’ in four environments

NO	Trait^a^	QTL	Environment	Chromosome	Position (cM)	Lod	Additive effect^b^	*R*^*2*^%^c^	Confidence interval (cM)	Flanking markers	Physical interval (Mbp)
1	GL	*qGL1.1*	2019	Ft1	39.6	6.74	0.114	8.39	38.2–43.9	Block332-Block411	6.43–8.63
			2017	Ft1	40.5	6.17	0.094	6.23			
			2018	Ft1	40.8	14.91	0.186	15.97			
2	GL	*qGL1.2*	2018	Ft1	49.0	6.33	0.127	7.40	48.8–51.5	Block442-Block506	9.61–11.14
3	GL	*qGL1.3*	2019	Ft1	77.8	6.35	0.126	9.81	77.0–78.2	Block747-Block765	18.89–19.86
4	GL	*qGL1.4*	2017	Ft1	87.6	28.64	0.235	39.43	87.3–88.6	Block892-Block911	23.13–23.71
			2018	Ft1	87.6	27.57	0.304	34.17			
			2019	Ft1	87.6	20.46	0.205	27.44			
5	GL	*qGL1.5*	2019	Ft1	93.1	12.66	0.168	18.40	91.5–94.3	Block935-Block969	24.35–25.18
6	GL	*qGL3.1*	2017	Ft3	163.7	4.77	-0.104	4.75	163.5–165.1	Block6544-Block6676	50.00–51.01
7	L/W	*qL/W1.1*	2020	Ft1	69.2	4.17	0.052	7.50	66.7–72.2	Block669-Block714	15.70–17.58
8	L/W	*qL/W1.2*	2017	Ft1	87.6	40.78	0.113	54.85	87.0–88.3	Block889-Block911	23.02–23.71
			2018	Ft1	87.6	43.59	0.132	58.79			
			2019	Ft1	87.6	35.52	0.110	49.36			
9	L/W	*qL/W1.3*	2018	Ft1	96.7	17.11	0.093	29.50	96.5–97.3	Block988-Block994	25.50–26.19
			2019	Ft1	96.7	16.36	0.083	27.25			
10	GW	*qGW1.1*	2017	Ft1	1.4	4.72	-0.035	6.32	0.0–2.5	Block2-Block8	0.17–0.47
11	GW	*qGW1.2*	2018	Ft1	6.9	4.47	-0.036	4.16	5.3–8.0	Block75-Block121	1.43–2.20
12	GW	*qGW1.3*	2018	Ft1	14.2	4.00	-0.035	3.75	12.8–14.4	Block196-Block256	3.87–4.81
13	GW	*qGW1.4*	2018	Ft1	20.9	4.05	-0.039	4.18	16.9–22.9	Block310-Block312	5.56–5.63
14	GW	*qGW1.5*	2019	Ft1	30.9	8.15	0.060	12.32	26.8–32.0	Block312-Block314	5.63–5.66
15	GW	*qGW1.6*	2020	Ft1	36.6	3.72	0.050	6.58	31.8–37.1	Block313-Block332	5.64–6.44
16	GW	*qGW1.7*	2017	Ft1	38.9	3.68	0.031	4.88	37.8–40.8	Block331-Block373	6.43–7.07
			2018	Ft1	38.9	25.49	0.098	29.70			
			2019	Ft1	38.9	13.95	0.069	17.92			
17	GW	*qGW1.8*	2020	Ft1	69.2	4.52	-0.056	8.06	68.2–71.9	Block673-Block715	16.08–17.63
18	GW	*qGW1.9*	2019	Ft1	81.4	6.58	-0.048	8.75	80.1–81.9	Block765-Block851	19.83–22.06
19	GW	*qGW1.10*	2018	Ft1	87.6	15.43	-0.073	16.18	86.3–88.8	Block882-Block914	22.39–23.79
			2017	Ft1	88.0	9.49	-0.053	14.26			
			2019	Ft1	88.0	11.22	-0.061	14.24			
20	GW	*qGW8.1*	2017	Ft8	0.0	4.65	0.035	6.15	0.0–3.3	Block14887-Block15042	2.00–7.35
			2018	Ft8	0.3	5.73	0.042	5.39			
21	GY	*qGY1.1*	2019	Ft1	35.1	6.00	133.075	10.36	32.3–35.7	Block314-Block321	5.66–6.42
22	GY	*qGY1.2*	2020	Ft1	37.6	6.45	461.016	10.56	36.2–46.5	Block324-Block428	6.42–9.15
			2017	Ft1	38.0	5.68	130.859	9.20			
			2019	Ft1	41.9	6.29	131.201	10.84			
			2018	Ft1	42.9	4.27	104.025	7.51			
23	GY	*qGY1.3*	2020	Ft1	47.4	4.99	392.254	9.03	46.8–48.5	Block427-Block442	9.10–9.68
24	GY	*qGY1.4*	2017	Ft1	163.8	3.37	-107.175	5.70	161.3–167.9	Block1140-Block1820	30.72–49.59
25	GY	*qGY1.5*	2017	Ft1	173.7	3.75	-104.918	5.94	170.0–174.1	Block1806-Block1869	49.02–50.91
26	GY	*qGY7.1*	2020	Ft7	65.1	3.30	-291.146	5.16	59.3–68.9	Block13743-Block13784	14.79–16.97
27	GY	*qGY8.1*	2020	Ft8	163.6	4.07	370.051	6.41	161.9–173.6	Block16245-Block16303	40.03–43.69
28	TGW	*qTGW1.1*	2017	Ft1	15.3	4.39	-0.641	4.96	14.9–22.9	Block263-Block312	5.03–5.63
			2018	Ft1	15.3	3.86	-0.582	3.41			
29	TGW	*qTGW1.2*	2020	Ft1	38.5	4.54	0.601	7.58	38.0–40.1	Block331-Block350	6.43–6.68
			2017	Ft1	38.9	18.07	1.372	23.58			
			2018	Ft1	38.9	37.06	2.147	47.51			
			2019	Ft1	38.9	18.21	1.206	24.45			
30	TGW	*qTGW4.1*	2019	Ft4	113.6	4.63	0.584	5.74	111.9–117.7	Block8426-Block8758	34.64–40.42
			2017	Ft4	117.0	4.67	0.674	5.81			
31	TGW	*qTGW4.2*	2019	Ft4	122.1	7.77	0.751	9.33	120.1–126.2	Block8778-Block8828	39.85–42.10
32	TGW	*qTGW4.3*	2019	Ft4	127.3	6.23	0.676	7.60	126.9–132.3	Block8828-Block8843	42.09–43.22
			2017	Ft4	131.3	7.63	0.856	9.20			

^a^*GY* Grain yield, *TGW* 1000-grains weight, *GL* Grain length, *GW* Grain width, *L/W* grain length-to-width ratio

^b^Positive and negative values indicate that favourable alleles are from 'Jinqiaomai 2' and 'Xiaomiqiao', respectively

^c^*R*^2^, phenotypic variance explained by the QTL

In detail, 7 QTLs for GY were detected on chromosomes Ft1, Ft7 and Ft8 with *R*^2^ values ranging from 5.16% to 10.84%. Among these QTLs, a major QTL for GY (*qGY1.2*), mapped between Block324 and Block428 on chromosome Ft1, was steadily detected in four environments with favourable allele derived from the high-yield parent ‘Jinqiaomai 2’, explaining 7.51%-10.84% of the phenotypic variance; the others were environment-specific QTLs (Table 3).

For TGW, 5 QTLs were identified on chromosomes Ft1, Ft4 and Ft8, explaining 5.06%-47.51% of the phenotypic variance, four of which were multi-environmental QTLs. A major QTL for TGW, *qTGW1.2*, mapped between markers Block331 and Block350 on chromosome Ft1, were consistently detected in four environments, explaining 7.58%-47.51% of the phenotypic variance. Three minor QTLs for TGW, *qTGW1.1*, *qTGW4.1* and *qTGW4.3*, were repeatedly detected in two environments, explaining 3.41%-4.96%, 5.74%-5.81% and 7.60%-9.20% of the phenotypic variance, respectively (Table 3). Expect for *qTGW1.1*, the favourable alleles of QTLs for TGW were all contributed by the large-grain parent ‘Jinqiaomai 2’.

For GL, 6 QTLs were identified on chromosomes Ft1 and Ft3, with *R*^2^ values ranging from 4.75% to 41.93%. Two major QTLs, *qGL1.1* and *qGL1.4*, mapped to Ft1 (Block332-Block411 and Block892-Block911) were repeatedly detected in four environments, with favourable alleles derived from the long-grain parent ‘Jinqiaomai 2’, explaining up to15.97% and 41.93% of the phenotypic variance, respectively. The other four QTLs were environment-specific QTLs (Table 3).

For GW, 11 QTLs were identified on chromosomes Ft1 and Ft8, with *R*^2^ values ranging from 3.75% to 29.70%, five of which were multi-environmental QTLs. Three major QTLs for GW, *qGW1.7*, *qGW1.9* and *qGW1.10*, mapped to Ft1 (Block331-Block373, Block765-Block851 and Block882-Block914), were consistently detected across three, two and four environments, explaining up to 29.70%, 11.43% and 16.82% of the phenotypic variance, respectively. Two minor QTLs, *qGW1.6* and *qGW8.1*, mapped to Ft1 (Block313-Block332 and Block673-Block715), were repeatedly detected in two environments, explaining 6.05%-6.58% and 5.39%-6.15% of the phenotypic variance, respectively (Table 3). Among the five stable QTLs for GW, favourable alleles of *qGW1.6*, *qGW1.7* and *qGW8.1* were contributed by long-grains parent ‘Jinqiaomai 2’, while favorable alleles of *qGW1.9* and *qGW1.10* were contributed by the short-grain parent ‘Xiaomiqiao’ (Table 3).

For L/W, 3 QTLs were identified on chromosome Ft1 (Block669-Block714, Block889-Block911 and Block988-Block994), with *R*^2^ values ranging from 7.50% to 58.79%. Two major QTLs for L/W, *qL/W1.2* and *qL/W1.3*, were consistently detected in two and four environments with favourable alleles contributed by ‘Jinqiaomai 2’, accounting for 49.36%-58.79% and 27.25%-29.50% of the phenotypic variation, respectively (Table 3).

### QTL cluster analysis

The 32 QTLs identified for the five grain-related traits were distributed on 24 chromosomal regions. The QTLs for different traits with an overlapping confidence interval were used to estimated the presence of QTL clusters. Finally, we obtained five QTL clusters on chromosome Ft1, which harboured 8 multi-environmental QTLs and 5 environment-specific QTLs (Fig. 1. and Table 4). One major QTL cluster, *qClu-1-3*, located between markers Block324 and Block428, harboured four stable major QTLs (*qGY1.2*, *qTGW1.2*, *qGW1.7* and *qGL1.1*) detected in at least three environments. Another major QTL cluster (*qClu-1-5*), mapped between markers Block882 and Block914, harboured three stable major QTLs (*qGL1.4*, *qL/W1.2* and *qGW1.10*) detected in three environments. QTL cluster *qClu-1-1* harboured one environment-specific QTL and one multi-environmental QTL repeatedly detected in two environments. QTL clusters *qClu-1-2* and *qClu-1-4* only harboured two environment-specific QTLs. Remarkably, *qClu-1-3* harboured QTLs controlling GY, TGW and grain size, and the elite alleles of QTLs were all from ‘Jinqiaomai 2’, indicating that the marker development from the genomic regions would be useful for marker-assisted selection (MAS) in the improvement of grain size and yield in Tartary buckwheat breeding.

**Table 4:** Five QTL clusters for grain weight/size detected in the XJ-RILs population derived from the cross of ‘Xiaomiqiao × Jinqiaomai 2’ in four environments

NO	**Cluster name**	Chromosome	Confidence interval (cM)	Flanking markers	Physical interval (Mbp)	QTL number	QTL
1	*qClu-1-1*	Ft1	14.9–22.9	Block263-Block312	5.03–5.63	2	*qTGW1.1*^#^, *qGW1.4*
2	*qClu-1-2*	Ft1	31.8–37.8	Block313-Block332	5.64–6.44	2	*qGY1.1*, *qGW1.6*^#^
3	*qClu-1-3*	Ft1	36.2–46.5	Block324-Block428	6.42–9.15	4	qGY1.2^*^, qTGW1.2^*^, *qGW1.7*^*&*^, qGL1.1^*^
4	*qClu-1-4*	Ft1	66.7–72.2	Block673-Block715	15.70–17.63	2	*qL/W1.1*, *qGW1.8*
5	*qClu-1-5*	Ft1	86.3–88.9	Block882-Block914	22.39–23.79	3	qGL1.4^*^, qL/W1.2^*^, qGW1.10^*^

^*^, & and # indicate QTLs repeatedly detected in four, three and two environments, respectively

### Candidate gene idenification in two major QTL clusters

The confidence intervals of two major QTL clusters, *qClu-1-3* and *qClu-1-5* for grain weight/size, were aligned to the current reference genome to identify the corresponding genes. *qClu-1-3* and *qClu-1-5* were positioned at 6.42–9.15 Mb and 22.39–23.79 Mb on chromosome Ft1 (Table 4), with 337 and 115 annotated genes, respectively (Additional file 1: Table S4). A total of 38 and 21 homologues of plant grain weight/size genes were found within the physical interval of *qClu-1-3* and *qClu-1-5*, respectively (Additional file 1: Table S5). These 59 putative candidate genes were homologues of 27 known grain weight/size genes (*LARGE8*/O*sMKP1*, *MKK4*, *OsMAPK6*, *SMG2*/*OsMKKK10*, *AHKs*, *ARF2*/*MNT*, *BRI1*, *D61*/*OsBRI1*, *DASH*, *GSE5*/*GW5*/*qSW5*, *GSK2*, *IKU2*, *PP2C-1*, *qGL3*/*GL3.1*/*OsPPKL1*, *TGW6*, *ZmGS5*, *ABI5*, *ANT*, *Awn-1*, *GS2*/*GL2*/*GLW2*/*PT2*, *LP1*, *MADS1*, *RPT2A*, *AGB1 ABA2*, *AGPase* and *CYP78A9*), involved in G-protein signalling, phytohormone signalling and homeostasis, mitogen-activated protein kinase (MAPK) signalling, the ubiquitin‒proteasome pathway, and some transcriptional regulators (Table 5).

**Table 5:** Homologous genes of plant grain weight/size-related genes in the physical intervals of the stable major QTL clusters of *qClu-1-3* and *qClu-1-5*

QTL cluster	Homologues of grain weight/size related genes of Tartary buckwheat	Plant grain weight/size genes	Protein category	Accession number
G-protein signalling				
*qClu-1-3*	*FtPinG0002488900.01*	*AGB1*	Gβ subunit	AT4G34460.4
*qClu-1-3*	*FtPinG0002472200.01*			AT4G34460.2
*qClu-1-3*	*FtPinG0002450600.01*			
Phytohormone signalling and homeostasis				
*qClu-1-3*	*FtPinG0002437500.01*	*AHKs*	Histidine kinases; cytokinin receptors	AT1G27320.1
*qClu-1-3*	*FtPinG0002474100.01*			AT2G01830.2
*qClu-1-3*	*FtPinG0002452300.01*			AT2G01830.3
*qClu-1-3*	*FtPinG0002469700.01*	*ARF2/MNT*	Auxin response factor	AT5G62000.3
*qClu-1-3*	*FtPinG0001423200.01*	*BRI1*	LRR-RLK; brassinosteroid receptor	AT4G39400.1
*qClu-1-3*	*FtPinG0002492200.01*			
*qClu-1-3*	*FtPinG0003208300.01*			
*qClu-1-5*	*FtPinG0001016500.01*			
*qClu-1-5*	*FtPinG0001017200.01*			
*qClu-1-5*	*FtPinG0001017300.01*			
*qClu-1-5*	*FtPinG0001017500.01*			
*qClu-1-5*	*FtPinG0001026100.01*			
*qClu-1-3*	*FtPinG0003206400.01*	*D61/OsBRI1*		Os01t0718300-02
*qClu-1-5*	*FtPinG0001023100.01*			
*qClu-1-3*	*FtPinG0002490200.01*	*DASH*	Endosperm-specific DOF transcription factor	Medtr2g014060
*qClu-1-3*	*FtPinG0002440300.01*	*GSE5/GW5/qSW5*	Calmodulin-binding protein	Os05t0187500-01
*qClu-1-3*	*FtPinG0002492500.01*	*GSK2*	SHAGGY-likekinase; regulator of brassinosteroid signaling kinase	Os05g020750
*qClu-1-3*	*FtPinG0002493000.01*	*IKU2*	LRR receptor kinase	AT3G19700.1
*qClu-1-3*	*FtPinG0002491700.01*			
*qClu-1-3*	*FtPinG0002451600.01*			
*qClu-1-5*	*FtPinG0001017700.01*			
*qClu-1-5*	*FtPinG0001023600.01*			
*qClu-1-5*	*FtPinG0001025300.01*			
*qClu-1-5*	*FtPinG0001010400.01*	*PP2C-1*	Phosphatase 2C-1	Glyma17g33690
*qClu-1-5*	*FtPinG0001027100.01*	*qGL3/GL3.1/OsPPKL1*	Protein phosphatase kelch family serine/threonine phosphatase	Os03t0646900-01
*qClu-1-3*	*FtPinG0002458000.01*	*TGW6*	IAA-glucosehydrolase	Os06t0623700-01
*qClu-1-3*	*FtPinG0002461400.01*	*ZmGS5*	Putative serine carboxypeptidase	GRMZM2G123815
Mitogen-activated protein kinase signalling pathway				
*qClu-1-3*	*FtPinG0002449100.01*	*LARGE8/OsMKP1*	Mitogen-activated protein kinase phosphatase	Os05t0115800-01
*qClu-1-5*	*FtPinG0001018400.01*	*MKK4*	Mitogen-activated protein kinase kinase	AT1G51660.1
*qClu-1-3*	*FtPinG0002471900.01*	*OsMAPK6*	Mitogen-activated protein kinase	Os06t0154500-01
*qClu-1-5*	*FtPinG0001026900.01*			
*qClu-1-3*	*FtPinG0002491900.01*	*SMG2/OsMKKK10*	Mitogen-activated protein kinase kinase kinase	Os04t0559800-01
*qClu-1-3*	*FtPinG0002491100.01*			
*qClu-1-3*	*FtPinG0002483600.01*			
*qClu-1-3*	*FtPinG0002464900.01*			
*qClu-1-3*	*FtPinG0002456200.01*			
*qClu-1-5*	*FtPinG0001016300.01*			
*qClu-1-5*	*FtPinG0001019400.01*			
*qClu-1-5*	*FtPinG0001025500.01*			
The ubiquitin–proteasome pathway				
*qClu-1-3*	*FtPinG0002435600.01*	*RPT2A*	26S proteasome regulatory particle AAA-ATPase	AT4G29040.1
*qClu-1-3*	*FtPinG0002435400.01*			
Transcriptional regulators				
*qClu-1-5*	*FtPinG0001029500.01*	*ABI5*	bZIP transcription factor	AT2G36270.1
*qClu-1-3*	*FtPinG0001427400.01*	*ANT*	AP2-like family transcription factor	AT4G37750.1
*qClu-1-5*	*FtPinG0001028600.01*			
*qClu-1-3*	*FtPinG0002470000.01*	*Awn-1 (An-1)*	Basic helix-loop-helix transcription factor	Os04t0350700-01
*qClu-1-5*	*FtPinG0001027800.01*			
*qClu-1-5*	*FtPinG0001033800.01*			
*qClu-1-5*	*FtPinG0001029100.01*	*GS2/GL2/GLW2/PT2*	OsGRF4	Os02g0701300
*qClu-1-3*	*FtPinG0001428200.01*	*LP1*	WRKY family transcription factor	Seita.2G369500.1.p
*qClu-1-3*	*FtPinG0002493300.01*			
*qClu-1-3*	*FtPinG0002461800.01*	*MADS1*	MADS-domain transcription factor	Os03t0215400-01
*qClu-1-3*	*FtPinG0002495300.01*			
Other regulators				
*qClu-1-3*	*FtPinG0002466600.01*	*ABA2*	Short-chain dehydrogenase/reductase	AT1G52340.1
*qClu-1-3*	*FtPinG0002473300.01*	*AGPase*	ADP-glucose pyrophosphorylase family protein	AT1G74910.2
*qClu-1-3*	*FtPinG0002490800.01*	*CYP78A9*	Cytochrome P450	AT3G61880.1
*qClu-1-3*	*FtPinG0002490600.01*			AT3G61880.2

### SNP/InDel variation analysis of candidate genes

Comparative genomics analysis between the two parents was carried out to identify the SNP/InDel variations in the physical intervals of the stable major QTL clusters *qClu-1-3* and *qClu-1-5*. The effects of the extracted SNPs/InDels were analysed (Additional file 1: Table S6). At the *qClu-1-3* interval, two genes showed at least one InDel variation, including one homologue of the plant grain weight/size gene (*FtBRI1*), and 23 genes showed at least one non-synonymous SNP variation between the two parents (Additional file 1: Table S7), including three homologues of plant grain weight/size genes, *FtPinG0002490200.01* (*FtDASH*), *FtPinG0002488900.01* (*FtAGB1*) and *FtPinG0002458000.01* (*FtTGW6*) (Table 6)*.* In addition, an SNP in intron 4 of *FtPinG0002495300.01*, a homologue of the grain weight/size regulator *MADS1*, led to alternative splicing. At the *qClu-1-5* interval, three genes had at least one non-synonymous SNP variation between the two parents (Additional file 1: Table S6), including two homologues of plant grain weight/size genes, *FtPinG0001018400.01* (*FtMKK4*) and *FtPinG0001028600.01* (*FtANT*) (Table 6).

**Table 6:** Annotation of non-synonymous SNP/InDel variations identified in homologues of plant weight/size genes within the physical interval of the stable major QTL clusters of *qClu-1-3* and *qClu-1-5*

QTL cluster	Chr	Homologues of grain weight/size related genes of Tartary buckwheat	Plant grain weight/size genes	Oritation	Transcript ID	Position bp	Exon	Mutation/Nucleotide	Mutation/Protein
*qClu-1-3*	Ft1	*FtPinG0001423200.01*	*BRI1*	-	FtPinG0001423200.01.T01	6,599,812	exon1:	c.1903-1906del	p.W635fs
*qClu-1-3*	Ft1	*FtPinG0002490200.01*	*DASH*	+	FtPinG0002490200.01.T01	6,985,415	exon1	c.C490G	p.H164D
*qClu-1-3*	Ft1	*FtPinG0002488900.01*	*AGB1*	-	FtPinG0002488900.01.T01	7,033,486	exon5	c.T635C; c.T221C	p.I212T; p.I74T
*qClu-1-3*	Ft1	*FtPinG0002458000.01*	*TGW6*	+	FtPinG0002458000.01.T01	8,026,188	exon3	c.C697G	p.Q233E
*qClu-1-5*	Ft1	*FtPinG0001018400.01*	*MKK4*	-	FtPinG0001018400.01.T01	23,023,413	exon1	c.A1311T	p.R437S
*qClu-1-5*	Ft1	*FtPinG0001028600.01*	*ANT*	-	FtPinG0001028600.01.T01	23,484,040	exon2	c.G521A	p.G174D

### qRT‒PCR analyses of candidate genes

**Fig. 2:**
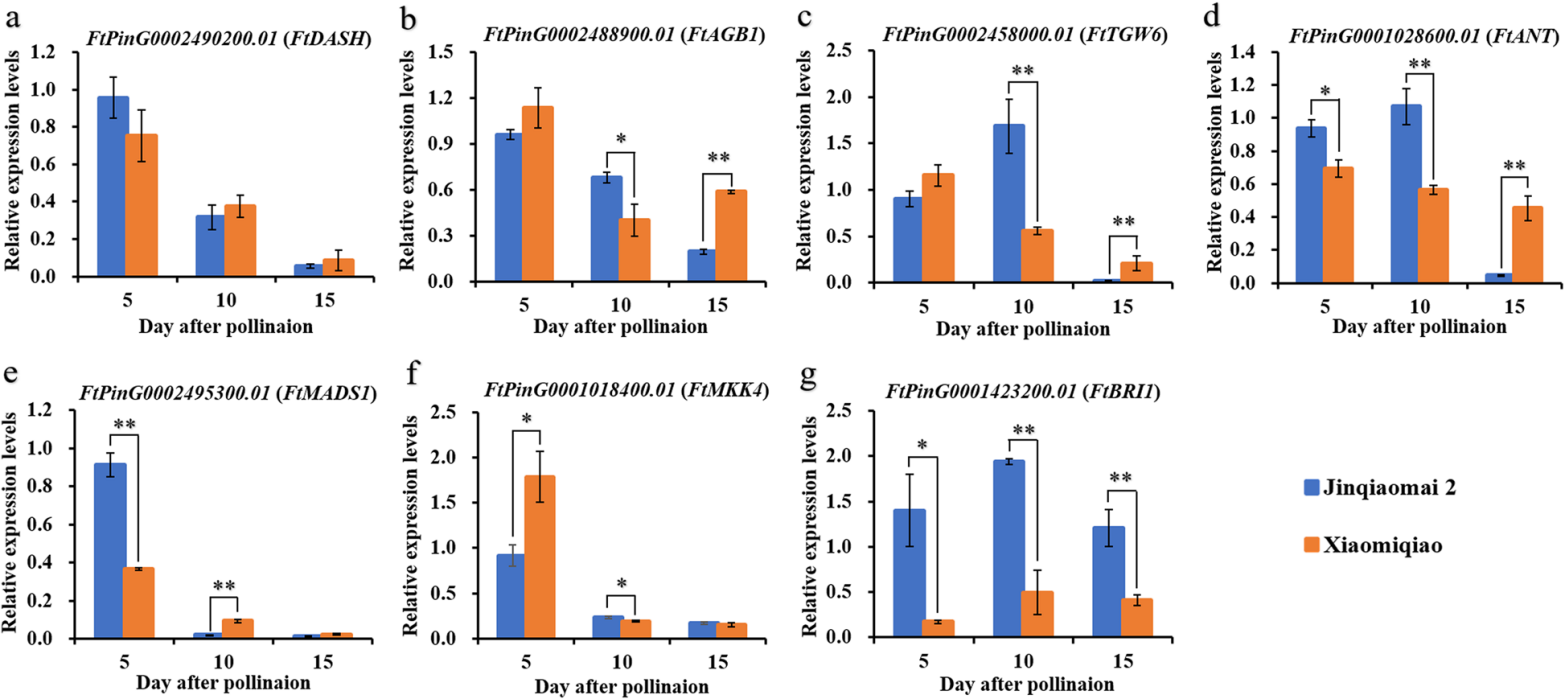
The expression of the seven homologues of grain weight/size genes in the parental lines Xiaomiqiao and Jinqiaomai 2

The expression levels of the seven homologues of known grain weight/size genes with SNP/InDel variations located on exons or splicing events were analysed in the two parents during grain development by qRT‒PCR. As shown in Fig. 2, all seven genes showed the lowest expression in the two parents at 15 days after pollination (DAP), expect *FtAGB1* and *FtBR1* in ‘Xiaomiqiao’, and significantly different expression was observed between the two parents during grain development stages, expect *FtADSH*. The expression levels of *FtAGB1* and *FtTGW6* in ‘Jinqiaomai 2’ were significantly or extremely significantly higher than those in ‘Xiaomiqiao’ at 10 DAP but lower than those in ‘Xiaomiqiao’ at 15 DAP (Fig. 2 b and c). *FtANT* and *FtBR1* showed significantly different expression levels between the two parents at 5, 10 and 15 DAP (Fig. 2 d and g). The expression level of *FtMADS1* in ‘Jinqiaomai 2’ was extremely significantly higher than that in ‘Xiaomiqiao’ at 5 DAP, but extremely significantly lower than that in ‘Xiaomiqiao’ at 10 DAP (Fig. 2 e). The expression level of *FtMKK4* in ‘Jinqiaomai 2’ was significantly lower than that in ‘Xiaomiqiao’ at 5 DAP but significantly higher than that in ‘Xiaomiqiao’ at 10 DAP (Fig. 2 f). This suggested that these six differential expression homologues could be key candidate genes for the grain development of Tartary buckwheat.

## Discussion

Genetic improvement in grain yield and quality has always been a permanent goal for Tartary buckwheat breeding. However, yield is a complex trait that is highly influenced by multiple yield-related traits and the environment. Evaluation of the yield-related traits of genetic populations or germplasms is an essential step in Tartary buckwheat breeding, genetic research and functional genomics research. In this study, the genetic variations in five grain-related traits, GY, TGW, GL, GW and L/W, were investigated in multiple environments using an RILs (XJ-RILs) population derived from a cross between two Tartary buckwheat varieties, Xiaomiqiao and Jinqiaomai2, who have large differences in yield, grain weight and grain size. A wide range and continuous distribution was observed for each of the five tested traits in the XJ-RILs population, suggesting that these traits were quantitative traits and controlled by multiple genes. The broad sense heritability of TGW and the three grain size traits, GL, GW and G/W, were over 79.5% and approximately two times higher than that of GY (37.8%), which was consistent with previous studies [17, 37], indicating that grain weight/size traits are relatively stable and less susceptible to environmental influences than GY. GY exhibited a significant positive correlation with TGW, and TGW showed strongly positive correlations with GL and GW and a significantly negative correlation with L/W. Thus, an increase in TGW and grain size has potential for Tartary buckwheat yield improvement. Moreover, higher Pearson's correlation coefficients were observed between TGW and GW than GL, which was in agreement with previous results in the bioparental population of Tartary buckwheat [17], suggesting that GW contributed more to the increase in TGW. The above mentioned results indicated that TGW and grain size with high heritability could be selected as indicators in Tartary buckwheat high-yield cultivar breeding; however, these traits cannot be selected alone due to the larger correlation among them.

QTL mapping for yield-related traits would provide a theoretical basis for functional gene discovery and molecular marker-assisted breeding of high-yield and high-quality Tartary buckwheat varieties. However, only a few QTLs/genes for yield-related traits have been identified in Tartary buckwheat until now. In this study, a high-density SNP linkage map of the XJ-RILs population developed from an earlier study result [31] was applied in QTL mapping. In total, 32 QTLs for five yield-related traits were detected in four environments located on chromosomes Ft1, Ft3, Ft4, Ft7 and Ft8, including 7 for GY, 5 for TGW, 6 for GL, 11 for GW and 3 for L/W. These QTLs were first identified, except for four QTLs for TGW (*qTGW1.1*, *qTGW1.2*, *qTGW4.1* and *qTGW4.2*) reported in our previous study [31]. Using GWASs, Zhang et al. [32] identified a candidate gene *FtPinG0404616900* (46,356,831–46,357,343 bp on chromosome Ft3) significantly affecting both TGW and GW and candidate genes *FtPinG0280000714* (29,286,254–29,289,3094 bp on Ft1) and *FtPinG0100980400* (2,824,410 to 2,825,743 bp on Ft4) associated with TGW and GW, respectively. However, these three candidate genes were not located in the physical region of QTLs identified in this study. Seven SSR markers were found to be associated with HGW (100-grain weight) by association analysis in two environments [38], among which SXAU1120, SXAU1130 and SXAU4246 were located in the physical region of the minor QTLs *qGL1.2*/*qGY1.3*, *qGY1.4* and *qTGW4.1*, respectively. Seven stable major QTLs obtained in this study were gathered into the QTL clusters *qClu-1-3* and *qClu-1-5* on chromosome Ft1. *qClu-1-3* spanning from 6.42 Mb to 9.15 Mb harboured four stable major QTLs, including *qGY1.2*, *qTGW1.2*, *qGW1.7* and *qGL1.1*. Several genes/loci underlying easy dehulling reported in previous studies [31, 39–41] were not collocated with the candidate genes/markers for grain weight/size identified by association analysis [32, 38], but were all located in the physical interval of *qClu-1-3*. This indicated that the genes underlying easy dehulling tended to have pleiotropism or physiological association with yield and grain weight/size, which can be further tested and verified by the fine mapping of *qClu-1-3* and will lay a theoretical foundation for breeding high-yield Tartary buckwheat varieties with large grains and easy dehulling using MAS. *qClu-1-5* spanning from 22.39 Mb to 23.79 Mb harboured three stable major QTLs for grain size (*qGL1.4*, *qL/R1.2* and *qGW1.10*). The two genomic regions may play an important role in regulating grain weight/size, which are of high importance in gene identificaton underling yield-related traits and marker development assisted breeding of high-yield Tartary buckwheat varieties.

Grain development is one of the most important determining factors of final grain yield and quality formation in cereal crops, which was also verified by the colocalization of QTLs for GY and grain weight/size in this study and previous reports [42, 43]. A better understanding of the molecular mechanisms of grain development and identification of genes related to grain weight/size will contribute to the improvement of crop yield and quality. To date, many genes regulating grain development have been identified, and several recent reviews have highlighted the possible molecular mechanisms and regulatory networks of grain weight/size control in model plants and crops [21, 23, 36]. The two parents of the mapping population in this study, ‘Xiaomiqiao’ and ‘Jinqiaomai 2’, were resequenced with approximately 20-fold coverage in our previous study [31]. Therefore, we combined QTL mapping, homology searches of known plant grain weight/size genes and comparative sequence analysis to predict and narrow the number of candidate genes within the physical interval of the two major QTL clusters detected in this study. A total of 59 candidate genes were identified as the homologues of 27 known plant grain weight/size genes within the physical interval of QTL clusters *qClu-1-3* and *qClu-1-5*, involving the mitogen-activated protein kinase (MAPK) signalling pathway (*LARGE8*/O*sMKP1*, *MKK4*, *OsMAPK6*, *SMG2*/*OsMKKK10*), phytohormone perception and homeostasis (*AHKs*, *ARF2*/*MNT*, *BRI1*, *D61*/*OsBRI1*, *DASH*, *GSE5*/*GW5*/*qSW5*, *GSK2*, *IKU2*, *PP2C-1*, *qGL3*/*GL3.1*/*OsPPKL1*, *TGW6*, *ZmGS5*), some transcriptional regulators (*ABI5*, *ANT*, *Awn-1*, *GS2*/*GL2*/*GLW2*/*PT2*, *LP1*, *MADS1*), the ubiquitin‒proteasome pathway (*RPT2A*), G-protein signalling (*AGB1*) and other regulators (*ABA2*, *AGPase*, *CYP78A9*) [21, 36]. *BRI1* encodes the brassinosteroid (BR) receptor BRASSINOSTEROID INSENSITIVE 1, and BRs were reported to positively regulate seed size in both Arabidopsis and rice [36, 44]. In this study, *FtBRI1* showed one InDel variation between the two parents, and the relative expression of *FtBRI1* in the large-grain parent ‘Jinqiaomai2’ was significantly higher than that in the small-grain parent ‘Xiaomiqiao’ at 5, 10 and 15 DAP, which was consistent with previous reports [36, 44]. Five homologues, *FtAGB1*, *FtANT*, *FtMKK4*, *FtDASH* and *FtTGW6*, showed at least one non-synonynous SNP variation between the two parents. In addition, one splicing event was identified in *FtMADS1*. *AGB1* encodes the G-protein β-subunit (Gβ), and *agb1-1* plants express similar fruit phenotype in *Arabidopsis* [45]. Loss-of-function or suppression of rice Gβ (*RGB1*) decreases grain size [46]. *ANT*, a member of the AP2-like family transcription factor, positively promotes seed and organ growth by mediating cell proliferation in *Arabidopsis* [47]. *MKK4* encodes a mitogen-activated protein kinase kinase and positively regulates grain size in rice [48]. Consistently, *FtAGB1*, *FtANT* and *FtMKK4* showed significantly or extremely significantly higher expression levels in the large-grain parent ‘Jinqiaomai 2’ than those in the small-grain parent ‘Xiaomiqiao’ at 10 DAP in this study. *MADS1* encodes a MADS-domain transcription factor, and a loss of function of *OsMADS1* causes splicing defects and leads to long grains [49]. MADS-box transcription factor genes have been shown to regulate growth and determine the easy dehulling of Tartary buckwheat grains [28, 40]. Consistently, the expression of *FtMADS1* in the long-grain parent ‘Jinqiaomai 2’ was extremely significantly lower than that in the short-grain and easy dehulling parent ‘Xiaomiqiao’ at 10 DAP in this study. *TGW6* encodes IAA-glucose hydrolase and negatively regulates endosperm development and grain weight in rice [50]. In contrast, the expression of *FtTGW6* in the large-grain parent ‘Jinqiaomai 2’ was extremely significantly higher than that in the small-grain parent ‘Xiaomiqiao’ at 10 DAP in this study. qRT‒PCR data indicated that the differential expression of *FtBRI1*, *FtAGB1*, *FtTGW6*, *FtANT*, *FtMKK4* and *FtMADS1* at the early grain development stage may lead to the grain weight/size difference between the two parents. Further studies are needed to elucidate the function of the six differential expression homologues in Tartary buckwheat grain weight/size control.

## Conclusion

In this study, we identified 32 QTLs for grain yield and grain weight/size distributed on 24 genomic regions in four environments using an RILs population. Two QTL clusters, *qClu-1-3* and *qClu-1-5*, located on chromosome Ft1, were revealed to harbour 7 stable major QTLs for yield and grain weight/size, which will promote marker development for high-yield breeding and gene fine mapping. Within the physical intervals of *qClu-1-3* and *qClu-1-5*, we searched 59 homologues of 27 known plant grain weight/size genes. Six homologues, *FtBRI1, FtAGB1, FtTGW6, FtANT, FtMKK4* and *FtMADS1*, with non-synonymous SNP /InDel variations and significantly differential expression between the two parents, may play important roles in Tatary buckwheat grain weight/size control and were selected as core candidate genes for further investigation.

## Supplementary Information

**Additional file 1:** **Table S1.** The weather and precipitation during the growth period in 2017, 2018, 2019 and 2020. **Table S2.** The primers sequences of candidate genes associated with grain weight/size and *actin* gene. **Table S3.** ANOVA and broad sense heritability of TGW, GL, GW, L/W and GY in the "Xiaomiqiao × Jinqiaomai 2" RILs population. **Table S4.** Genes located in the physical interval of the stable major QTL clusters *qClu-1-3* and *qClu-1-5*. **Table S5.** Homologues of plant grain weight/size genes in the physical intervals of the stable major QTL clusters *qClu-1-3* and *qClu-1-5*. **Table S6.** The categorization of SNPs and Indels within the physical interval of stable major QTL clusters *qClu-1-3* and *qClu-1-5*. **Table S7.** Non-synonymous SNP variations in exons and their effect in the physical interval of the stable major QTL clusers *qClu-1-3* and *qClu-1-5*.

**Additional file 2:** **Figure S1.** Violin plot of five grain-related traits in the ‘Xiaomiqiao × Jinqiaomai 2’ RILs population in four environments. GY, grain yield; TGW,1000-grain weight; GL, grain length; GW, grain width; L/W, grain length-width ratio. The shape of each violin indicates the probability density of the trait.

## Data Availability

All data generated or analyzed during this study are included in the main text article and its supplementary files.

## Abbreviations

QTL: Quantitative trait locus
GY: Grain yield
RILs: Recombinant inbred lines
TGW: 1000-Grain weight
GL: Grain length
GW: Grain width
L/W: Grain length-to-width ratio
DAP: Days after pollination
