# Supplementary figures and images for: QTL mapping and candidate gene analysis for yield and grain weight/size in Tartary buckwheat
Source: BMC Plant Biol. 2023 Jan 26;23:58. doi: 10.1186/s12870-022-04004-x (PMC9878770; doi:10.1186/s12870-022-04004-x)

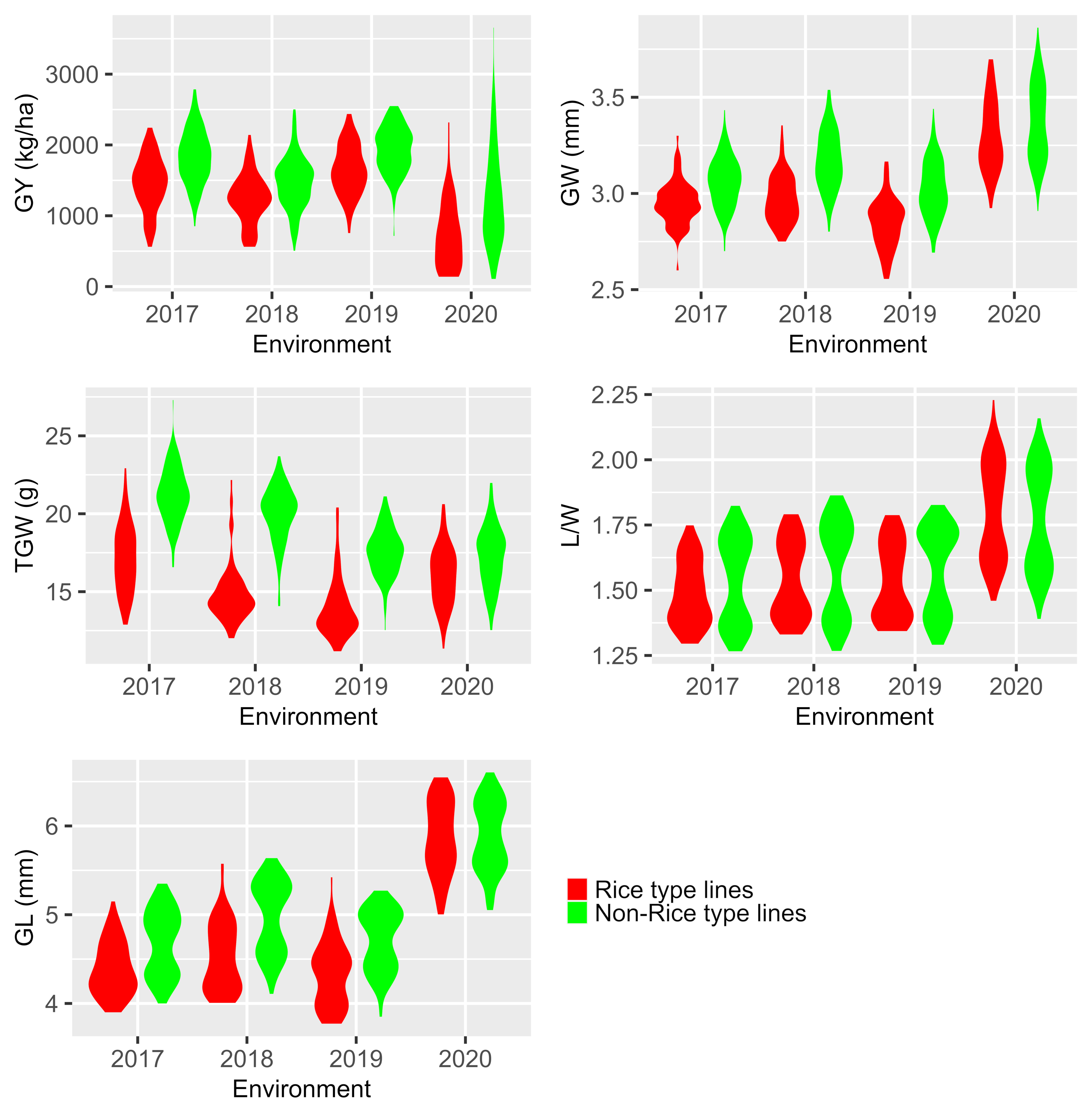

Supplement: Supplementary file 2 — Additional file 2: Figure S1. Violin plot of five grain-related traits in the ‘Xiaomiqiao × Jinqiaomai 2’ RILs population in four environments. GY, grain yield; TGW,1000-grain weight; GL, grain length; GW, grain width; L/W, grain length-width ratio. The shape of each violin indicates the probability density of the trait. [file 12870_2022_4004_MOESM2_ESM.jpg]
